# Large-Scale Spatial Distribution Patterns of Echinoderms in Nearshore Rocky Habitats

**DOI:** 10.1371/journal.pone.0013845

**Published:** 2010-11-05

**Authors:** Katrin Iken, Brenda Konar, Lisandro Benedetti-Cecchi, Juan José Cruz-Motta, Ann Knowlton, Gerhard Pohle, Angela Mead, Patricia Miloslavich, Melisa Wong, Thomas Trott, Nova Mieszkowska, Rafael Riosmena-Rodriguez, Laura Airoldi, Edward Kimani, Yoshihisa Shirayama, Simonetta Fraschetti, Manuel Ortiz-Touzet, Angelica Silva

**Affiliations:** 1 School of Fisheries and Ocean Sciences, University of Alaska Fairbanks, Fairbanks, Alaska, United States of America; 2 Department of Biology, University of Pisa, Pisa, Italy; 3 Departamento de Estudios Ambientales, Centro de Biodiversidad Marina, Universidad Simon Bolivar, Caracas, Venezuela; 4 Atlantic Reference Centre, Huntsman Marine Science Centre, St. Andrews, New Brunswick, Canada; 5 Department of Zoology, University of Cape Town, Rondebosch, South Africa; 6 Bedford Institute of Oceanography, Dartmouth, Nova Scotia, Canada; 7 Department of Biology, Suffolk University, Boston, Massachusetts, United States of America; 8 Marine Biological Association of the United Kingdom, Plymouth, United Kingdom; 9 Centro Interdipartimentale di Ricerca per le Scienze Ambientali, University of Bologna, Ravenna, Italy; 10 Kenya Marine and Fisheries Research Institute, Mombasa, Kenya; 11 Programa de Investigación en Botánica Marina, Departamento de Biologia Marina, Universidad Autónoma de Baja California Sur, La Paz, Baja California Sur, México; 12 Seto Marine Biological Laboratory, Kyoto University, Wakayama, Japan; 13 Laboratorio di Zoologia e Biologia Marina, Dipartimento di Scienze e Tecnologie Biologiche ed Ambientali, Università del Salento, Lecce, Italy; 14 Centro de Investigaciones Marinas, Universidad de La Habana, Miramar Playa, Ciudad de La Habana, Cuba; National Institute of Water & Atmospheric Research (NIWA), New Zealand

## Abstract

This study examined echinoderm assemblages from nearshore rocky habitats for large-scale distribution patterns with specific emphasis on identifying latitudinal trends and large regional hotspots. Echinoderms were sampled from 76 globally-distributed sites within 12 ecoregions, following the standardized sampling protocol of the Census of Marine Life NaGISA project (www.nagisa.coml.org). Sample-based species richness was overall low (<1–5 species per site), with a total of 32 asteroid, 18 echinoid, 21 ophiuroid, and 15 holothuroid species. Abundance and species richness in intertidal assemblages sampled with visual methods (organisms >2 cm in 1 m^2^ quadrats) was highest in the Caribbean ecoregions and echinoids dominated these assemblages with an average of 5 ind m^−2^. In contrast, intertidal echinoderm assemblages collected from clearings of 0.0625 m^2^ quadrats had the highest abundance and richness in the Northeast Pacific ecoregions where asteroids and holothurians dominated with an average of 14 ind 0.0625 m^−2^. Distinct latitudinal trends existed for abundance and richness in intertidal assemblages with declines from peaks at high northern latitudes. No latitudinal trends were found for subtidal echinoderm assemblages with either sampling technique. Latitudinal gradients appear to be superseded by regional diversity hotspots. In these hotspots echinoderm assemblages may be driven by local and regional processes, such as overall productivity and evolutionary history. We also tested a set of 14 environmental variables (six natural and eight anthropogenic) as potential drivers of echinoderm assemblages by ecoregions. The natural variables of salinity, sea-surface temperature, chlorophyll *a*, and primary productivity were strongly correlated with echinoderm assemblages; the anthropogenic variables of inorganic pollution and nutrient contamination also contributed to correlations. Our results indicate that nearshore echinoderm assemblages appear to be shaped by a network of environmental and ecological processes, and by the differing responses of various echinoderm taxa, making generalizations about the patterns of nearshore rocky habitat echinoderm assemblages difficult.

## Introduction

Biodiversity assessments in marine systems are of great interest from ecological, public and management standpoints. They are important for understanding ecological patterns and ecosystem functioning and for managing marine resource use and identifying conservation priorities [1]–[3]. A particular ecological interest is the identification of large-scale biodiversity patterns to investigate possible factors driving diversity, and to serve as context for local ecological studies and in management and conservation [4]. It has long been postulated that diversity in marine species or communities may follow latitudinal gradients with diversity peaking at the equator and declining towards higher latitudes [5], with evolutionary, historical and ecological mechanisms suggested as drivers [6]. Support for this trend is evident from shallow waters to the deep-sea [7]–[10] and a recent meta-analysis suggests that the trend can be viewed as a generalized pattern in marine taxa [11]. Nevertheless, biodiversity in some taxa or communities does not follow this postulated general latitudinal gradient. While this gradient may be an overarching feature, there are notable exceptions, e.g., in macroalgae [12]–[14], in benthic soft-sediment shelf communities [14]–[16] and rocky intertidal communities [17]. As intriguing as the idea of a generalized diversity pattern in marine communities may be, it is equally important to better understand large-scale diversity patterns for individual taxa and habitat types. This will allow for the development and further hypothesis testing needed to explain latitudinal and other large-scale marine biodiversity patterns [11], [18]–[20].

No global assessments of echinoderm diversity exist despite the often critical ecological roles they fulfill in various ecosystems worldwide. For example, holothurians can be highly diverse and are the dominant megabenthic taxon in some deep-sea systems, where they are critical in bioturbation and redistribution of fresh phytodetritus deposits [21]–[22]. On Arctic shelf systems, ophiuroids are the dominant taxon that accounts for a large portion of remineralization [23]–[24]. In several temperate nearshore, kelp-dominated systems, sea urchins have a keystone species function where their grazing activity switches the system between alternate stable states of lush kelp beds/macroalgae and urchin barrens [25]–[27]. Similarly, the asteroid *Pisaster ochraceus* in the Pacific Northwest is a keystone predator in the rocky intertidal with its feeding activity maintaining a diverse community and preventing mussels from out-competing other space occupiers [28]–[29]. Also, ecosystem structure and diversity of coral reefs can be strongly influenced by grazing and predation activities of the sea urchin *Diadema antillarum* in the Caribbean [30]–[31] and the crown-of-thorn sea star *Acanthaster planci* in the Australian Great Barrier Reef [32].

It is not always a single echinoderm species or class that contributes to overall ecosystem functioning. Rather, high echinoderm species numbers and abundances contribute significantly to community structure in different regions of the world. Examples of abundant and diverse echinoderm assemblages are reported from the nearshore regions of the Colombian Pacific coast [33], at Mauritius in the Indian Ocean [34], the Galapagos Islands in the Pacific [35], coasts along the tropical west Pacific [36], the nearshore regions of the Alaska Pacific coast [37], and in the Atlantic shelf benthos around the British Isles [38]. Despite this regional knowledge of echinoderms, there are surprisingly few large-scale studies analyzing echinoderm distribution and assemblage patterns.

The purpose of this study was to increase our understanding of echinoderm large-scale distribution and diversity patterns, and to identify possible drivers that may influence any observed patterns. The focus was on rocky intertidal and shallow rocky subtidal habitats. The nearshore zone is ecologically important as a highly productive region that provides important ecosystem goods and services [2], [39]–[43], but it is also most impacted and used by humans [44]–[47], with about 60% of the world population living along coasts and bays [48]. We used globally-distributed data from rocky nearshore areas collected with a standardized sampling protocol to: 1) examine possible trends in echinoderm abundance and diversity among ecoregions and with latitude, and 2) identify if there are common environmental drivers that may explain large-scale patterns in echinoderm assemblages.

## Methods

**Figure 1:**
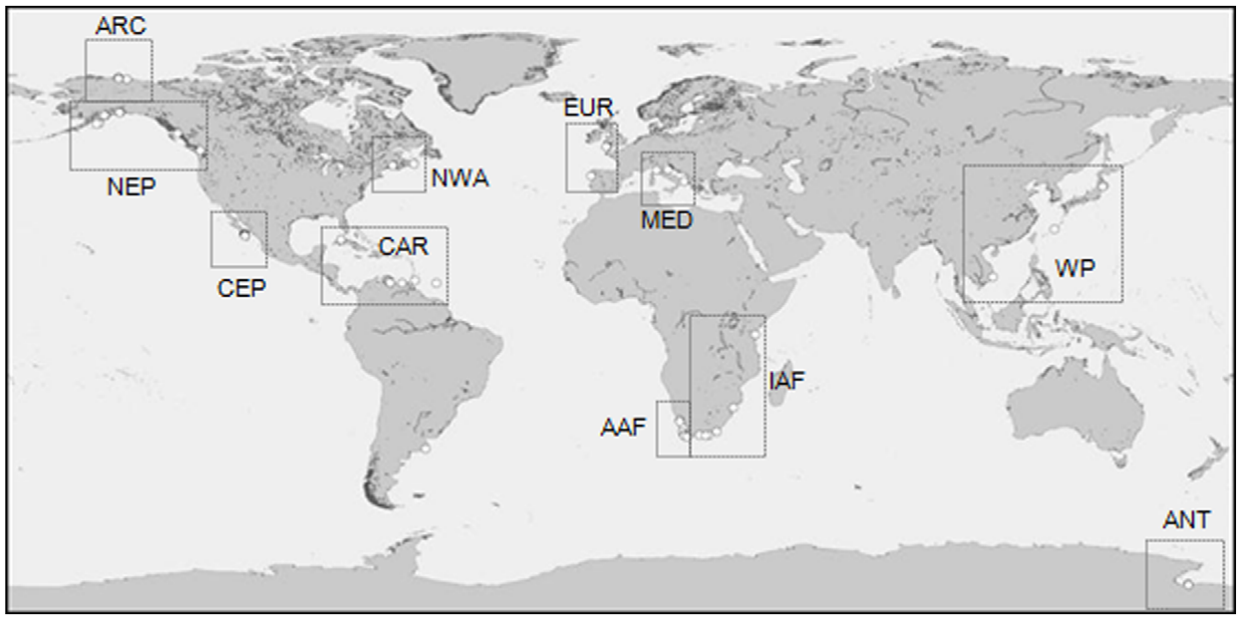
Global distribution of 76 sampling sites of echinoderm assemblages within the NaGISA program. Due to the large scale of the map, spatially close sites cannot be distinguished. Boxes delineate ecoregions (see text for details).

Echinoderms were collected following the standardized protocols of the Census of Marine Life NaGISA program (Natural Geography in Shore Areas, www.coml.nagisa.org) for coastal hard substrate sites with macroalgal cover (from here on referred to as “macroalgal habitats”) [49]. A total of 76 rocky macroalgal habitat sites were sampled between 2003 and 2009, with the majority of sampling occurring between 2006–2008 (Supplementary Table S1). Only data for one year per site at a time of highest community development were included. Sites were globally but not evenly distributed across the world's shores (Fig. 1). Position of sites depended much on accessibility and on location of contributing investigators. In general, more sites were sampled in the northern than in the southern hemisphere, and more sites in the western than in the eastern hemisphere. Several regions of the world's coastline were poorly sampled (e.g. Asia) or not at all sampled (e.g. Australia). Sites were selected based on relatively pristine conditions and remote from direct human influence as much as possible.

Sites were typically sampled at the high, mid and low intertidal levels and at 1 m, 5 m, 10 m and occasionally 15 m subtidal depth contours. Along each stratum, five replicate 0.0625 m^2^ quadrats (from hereon referred to as 16x) were placed at randomly selected (random number calculator) locations but with at least 1 m distance between adjacent replicates. All epifaunal invertebrates were removed from the quadrat area and echinoderms were identified to the lowest taxonomic level possible (typically species or genus). At several sites, echinoderms were enumerated using 1 m^2^ quadrats (from hereon referred to as 100x) either in addition to or instead of the 16x quadrats (Supplementary Table S1). In the 100x quadrats, all visible echinoderms >2 cm were counted without removal of the epifauna. Of the 76 macroalgal habitat sites sampled, 12 were sampled only with 16x quadrats, 34 were sampled only with 100x quadrats, and 30 sites were sampled with both quadrat sizes (Supplementary Table S1). Data from the two quadrat sizes were analyzed separately for all sites, resulting in four assemblage types considered: 16x intertidal, 16x subtidal, 100x intertidal, and 100x subtidal.

Local constraints prevented the sampling of all depth strata at all sites. Abundance data were therefore averaged for intertidal (high-low intertidal) and subtidal (1–15 m depths) regimes. Taxon richness, a basic diversity measure, is particularly sensitive to sampling effort with a higher likelihood to encounter more taxa with increased sampling [50]. To appropriately address this problem we used sample-based rarefaction to calculate the expected number of taxa for five quadrats (ES_5_) at each site for intertidal and subtidal assemblages [50]–[52]. Taxonomic distinctness (Δ*), a diversity measure that is largely independent of sampling effort and absolute abundances [53], was also obtained. This index ranges from 0 to 100, allocating distances based on the taxonomic level at which two taxa are related. Taxonomic levels included in the analysis were species, genus, family, order, and class, with equal weighting factors applied among all taxonomic levels (Primer-E v6 software).

For large-scale comparisons of echinoderm abundance and taxon richness (based on ES_5_ per site), sites were grouped into eco-regions based on geography and prevailing oceanographic conditions: Alaskan Arctic (ARC), north-east Pacific (NEP), central-east Pacific (CEP), Mediterranean (MED), European Atlantic (EUR), north-west Atlantic (NWA), Caribbean (CAR), Indian Ocean Africa (IAF, warm Agulhas current influence), Atlantic Ocean Africa (AAF, cold Benguela current influence), Antarctic McMurdo Sound (ANT) (see Fig. 1 and Supplementary Table S1). Regions with less than at least three sites for each quadrat size/tidal regime combination were excluded (e.g., Western Pacific Asia, Atlantic South America). We caution that all ecoregions likely were under-sampled to be a true representation of that region, but this grouping allowed for some preliminary comparisons of larger-scale patterns above the local variability. We therefore did not statistically compare abundance and richness in ecoregions but we offer descriptive trends on abundance and species composition and richness for ecoregions. Relationships of abundance, ES_5_ and taxonomic distinctness with latitude were analyzed using non-parametric Spearman Rank Correlations (SPSS). To extract the effect of sampling effort (i.e., number of quadrats sampled per intertidal or subtial regime at each site) on intertidal and subtidal abundance, in addition quadrat numbers were regressed against abundance and the residuals were used in correlations with latitude [54].

A set of 14 environmental drivers available for 54 sites within ecoregions (Supplementary Table S1) were correlated with biological site data pooled by ecoregion. We grouped environmental variables into six “natural” and eight “anthropogenic” variables (Table 1). Natural environmental drivers included: substrate type (SUB), macroalgal biomass (ALG), sea surface temperature (SST), chlorophyll *a* (CHA), primary productivity (PP), and salinity (SAL). Indices of anthropogenic variables of inorganic pollution (INP), organic pollution (ORP), nutrient contamination (NUTC), marine-derived pollution (MARP), acidification (AC), invasive species incidence (INV), human coastal population density (HUM), and shipping activity (SH) were taken from 1 km resolution global models of human impacts on marine ecosystems by Halpern et al. [55] (Table 1).

**Table 1:** List of environmental variables used in analysis.

Variable	Short	Description	Reference
**Natural**			
Macroalgal biomass	ALG	Macroalgal wet weight per unit area	NaGISA data
Substrate category	SUB	Categories: bedrock, sandstone, large boulders, boulders and cobbles, rocks embedded in soft sediment	NaGISA data
Sea-surface temperature	SST	climatological summer mean value, averaged between 1985 and 2001, derived from the 4 km resolution AVHRR Pathfinder Project version 5.0 by the NOAA NODC	[125]
Chlorophyll-*a*	CHA	SeaWiFS reprocessing 5.2 by the NASA GSFC Ocean Color Group, averaged 1997-2009, 9 km resolution	[126]
Primary productivity	PP	mg carbon m^−2^ d^−1^, Vertically Generalized Production Model (VGPM) for SeaWiFS, averaged 1997–2007, 18 km resolution	[127]
Salinity	SAL	HYCOM predictive model, National Ocean Partnership Program, average for 2003, 1/12° resolution	http://www.hycom.org/
**Anthropogenic**			
Inorganic pollution	INP	urban runoff estimated from land-use categories, US Geologic Survey (http://edcsns17.cr.usgs.gov/glcc/)	[55]
Organic pollution	ORP	FAO national pesticides statistics (1992–2002), (http://faostat.fao.org)	[55]
Nutrient contamination	NUTC	FAO national fertilizers statistics (1992–2002), (http://faostat.fao.org)	[55]
Marine-derived pollution	MARP	port data 1999–2005, proportional to commercial shipping traffic	[55]
acidification	AC	aragonite saturation state 1870–2000/2009, 1 degree lat/long resolution	[55]
invasive species incidence	INV	cargo traffic 1999–2003	[55]
human coastal population density	HUM	LandScan 30 arc-second population data of 2005	[55]
shipping activity	SH	commercial ship traffic 2004–2005	[55]

For the biological-environmental comparison we pooled samples for the two tidal regimes (intertidal and subtidal) per site and averaged both biological and environmental data for ecoregions. Data for the quadrat sizes of 16x and 100x were maintained separately. Only ecoregions that contained at least three sites where biological and environmental data were available were included in the analysis. We used ecoregions as the scale of comparison because of the potential inaccuracy of satellite-derived data from optical sea-surface properties (e.g., chlorophyll-a, primary productivity) on small spatial scales [55]. Different resolution of our biological data collected at a scale of 10's of meters (quadrats within the intertidal and subtidal regime of a specific site) and environmental data extracted from global models can be problematic. Although anthropogenic variables were sampled at a 1 km resolution, the nearshore environment is highly variable and can be under the influence of point sources. By combining site data for ecoregions we concentrate on large-scale variability, which exceeds the small-scale, local variability where the above-mentioned uncertainties are most profound [56]–[57].

Biological communities over large spatial scales often do not share any common species; hence, we used the taxonomic dissimilarity coefficient Theta instead of the commonly-used Bray-Curtis index for multivariate comparisons. Theta is based on the presence/absence of species and the taxonomic relationship (class to species used as taxonomic levels) of species within each sample [58]. Theta dissimilarity matrices by ecoregions were utilized to determine which environmental variables (also by ecoregions) are most influential on echinoderm assemblages using the BEST Bio-Env procedure within Primer-E. Only those sites where both biological and environmental data were available were included in the pooling of ecoregions and analyses (Supplementary Table S1). Environmental variables were normalized to create a common, dimensionless measurement scale and examined for correlation prior to analysis using Spearman Rank correlations. None of the variables were correlated at rho≥0.95 and thus all variables were maintained in the analyses. Analyses were conducted including all environmental variables and, if variables derived from satellite data (CHL and PP) were identified as drivers, we repeated the analysis by excluding these variables to assess any biases occurring from these data sources.

## Results

### Ecoregional patterns in echinoderm abundance and diversity

**Figure 2:**
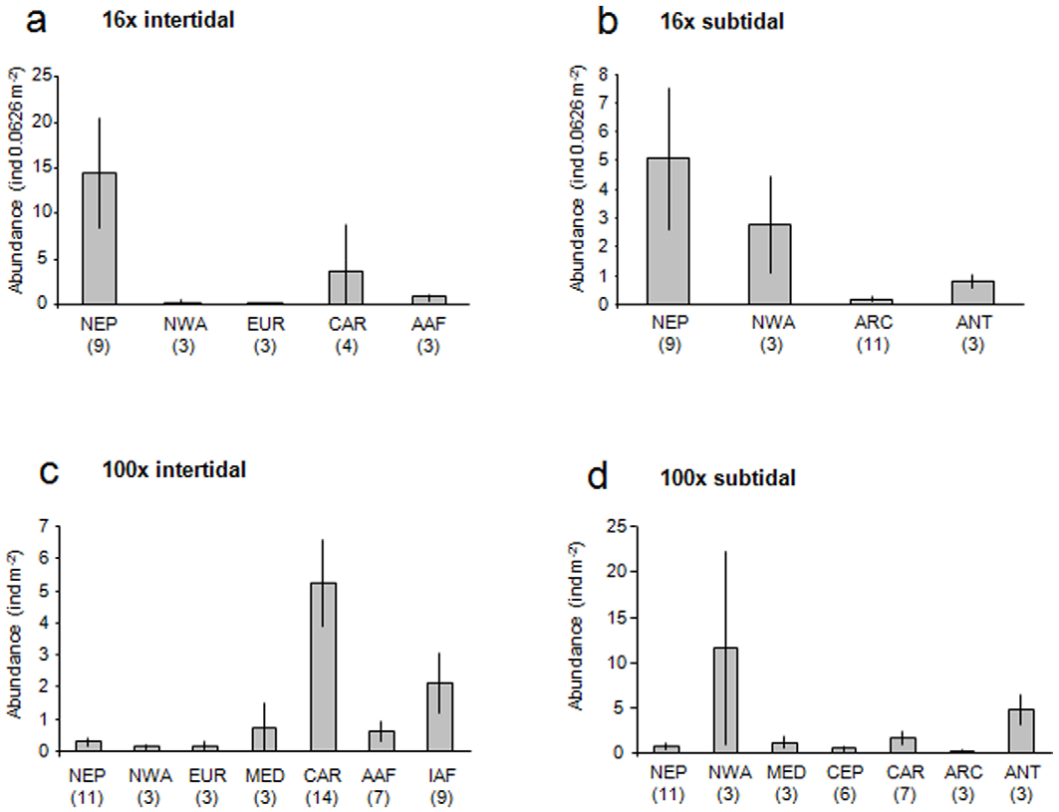
Average echinoderm abundances in ecoregions. **a.** 16x intertidal collections, **b**. 16x subtidal collections, **c.** 100x intertidal collections, **d.** 100x subtidal collections. Numbers below ecoregions specify the number of sites included in each region. See Fig. 1 and text for ecoregions.

**Figure 3:**
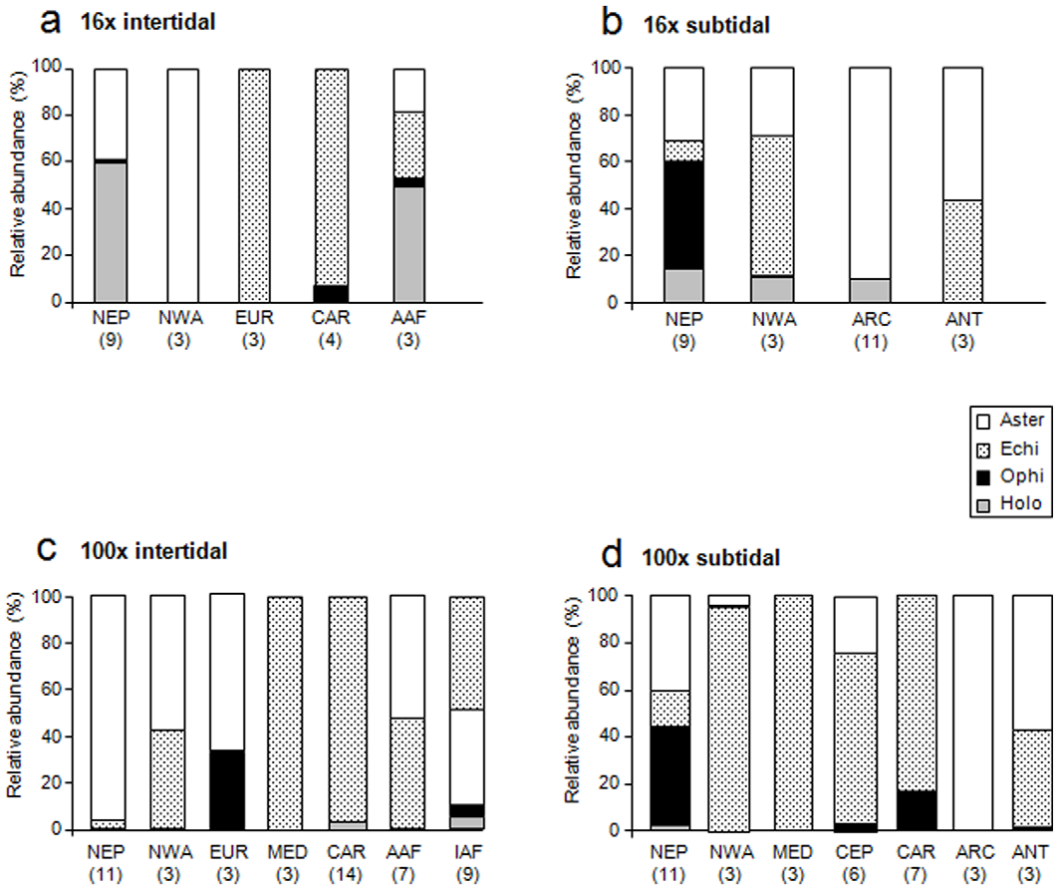
Relative abundances of echinoderm classes in ecoregions. **a.** 16x intertidal collections, **b.** 16x subtidal collections, **c.** 100x intertidal collections, **d.** 100x subtidal collections. Numbers below ecoregions specify the number of sites included in each region. See Fig 1 and text for ecoregions.

A total of 86 echinoderm taxa were found across all sites, tidal regimes and quadrat sizes; among these were 32 asteroids, 18 echinoids, 21 ophiuroids, and 15 holothuroids. In most ecoregions variability among sites was high. Within intertidal assemblages collected with 16x quadrats, highest abundances were found in the Northeast Pacific (mean±se 14.4±5.9 ind 0.0625 m^−2^) and the Caribbean (3.9±3.7 ind 0.0625 m^−2^) (Fig. 2a). In all other regions, average abundance was less than 1 ind 0.0625 m^−2^; no echinoderms were found in the intertidal at only one site sampled in the Mediterranean (data not shown). The abundant intertidal assemblages in the Northeast Pacific were dominated by asteroids and holothurians (Fig. 3a), represented nearly exclusively by *Leptasterias* spp. and *Cucumaria vegae*, respectively. In the Caribbean, mostly echinoids dominated by the two species, *Echinometra viridis* and *E. lucunter*. Average abundances were also high in the Northeast Pacific (5.1±2.5 ind 0.0625 m^−2^) in the 16x subtidal assemblages, but were not significantly different from those in the Northwest Atlantic (2.8±1.7 ind 0.0625 m^−2^) (Fig. 2b). Abundances in both polar regions were less than 1 ind 0.0625 m^−2^. The abundant subtidal assemblages in the Northeast Pacific consisted of a variety of species within four echinoderm classes (Fig. 3b): asteroids (mostly *Pycnopodia helianthoides*, *Evasterias troshelii*, *Henricia leviuscula*, *Leptasterias* spp., and *Orthasterias koehleri*), echinoids (*Strongylocentrotus droebachiensis*), ophiuroids (mostly *Amphipholis* spp. and *Ophiopholis aculeata*) and holothuroids (mostly *Cucumaria vegae*). In the abundant 16x subtidal assemblages in the Northwest Atlantic, asteroids were dominated by *Asterias* spp., echinoids by *S. droebachiensis*, and ophiuroids by *O. aculeata*.

For intertidal assemblages sampled with the larger (100x) quadrats, abundance was highest in the Caribbean (mean±se 5.2±1.3 ind m^−2^) and on the African Indian Ocean coast (2.1±1.0 ind m^−2^), and was less than 1 ind m^−2^ in all other ecoregions (Fig. 2c). No echinoderms occurred at the two sites collected in the West Pacific (data not shown). Echinoids dominated in the Caribbean (Fig. 3c), specifically with *Echinometra viridis* and *E. lucunter* and occasionally *Diadema antillarum*. All four classes occurred in the African Indian Ocean but were dominated by asteroids (mostly *Patiriella exigua*) and echinoids (especially *Parechinus angulosus* and *Echinometra mathaei*). Abundances were not significantly different among ecoregions in the subtidal assemblages from 100x quadrats, likely due to high variability among sites within the Northwest Atlantic region, where abundance was highest (11.6±10.7 ind m^−2^) (Fig. 2d). Assemblages there were dominated by the echinoid *Strongylocentrotus droebachiensis* (Fig. 3d).

**Figure 4:**
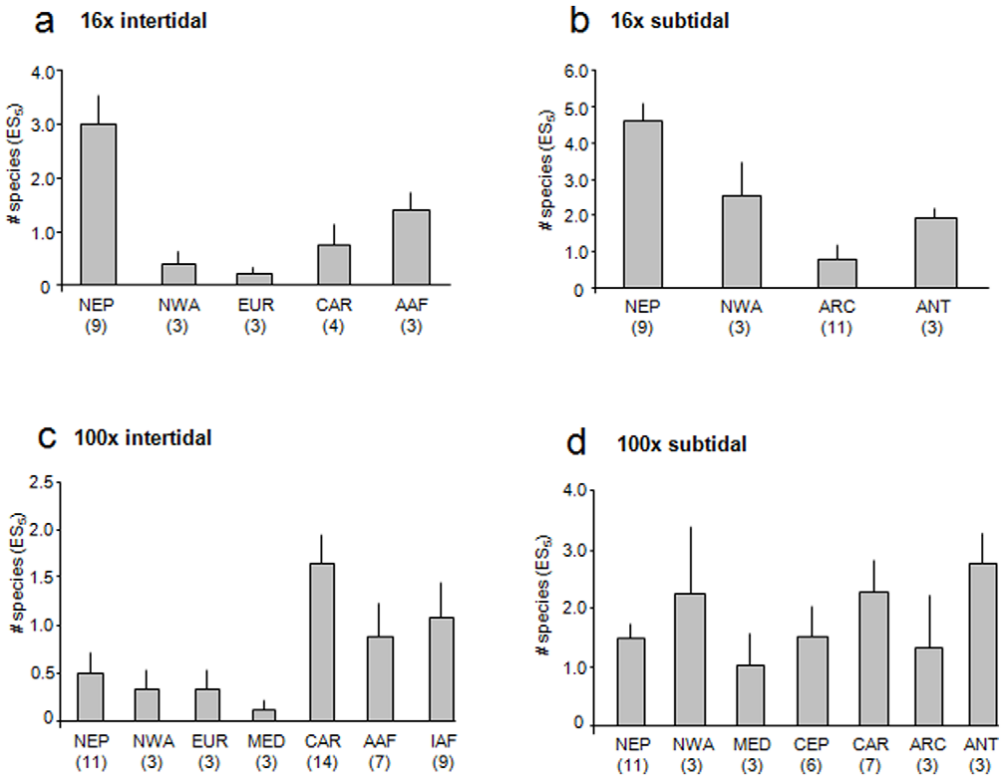
Expected number of species (ES_5_) in ecoregions. **a.** 16x intertidal collections, **b.** 16x subtidal collections, **c.** 100x intertidal collections, **d.** 100x subtidal collections. Numbers below ecoregions specify the number of sites included in each region. See Fig. 1 and text for ecoregions.

Species richness (ES_5_) for all regions and tidal regimes was rather low. Intertidal assemblages from 16x quadrat collections were most species-rich in the Northeast Pacific (mean±se 3.0±0.5 ES_5_) and along the Atlantic Ocean African coast (1.4±0.4) (Fig. 4a). ES_5_ in all other ecoregions was less than 1. Similarly to the intertidal regime, species richness for subtidal 16x assemblages was also highest in the Northeast Pacific (4.6±0.5 ES_5_), followed by the Northwest Atlantic (2.6±0.9 ES_5_), and the Antarctic McMurdo Sound (1.9±0.3 ES_5_) (Fig. 4b). In intertidal assemblages collected with the 100x quadrats, species richness was highest in the Caribbean (1.6±0.3 ES_5_), followed by the African Indian and Atlantic Ocean coasts (1.1±0.4 and 0.9±0.4 ES_5_, respectively) (Fig. 4c). Species richness in subtidal 100x assemblages was similar among ecoregions with highest values in the Antarctic McMurdo Sound (2.8±0.3 ES_5_) and lowest in the Mediterranean (1±0.5 ES_5_) (Fig. 4d).

### Latitudinal trends in echinoderm abundance and diversity

**Figure 5:**
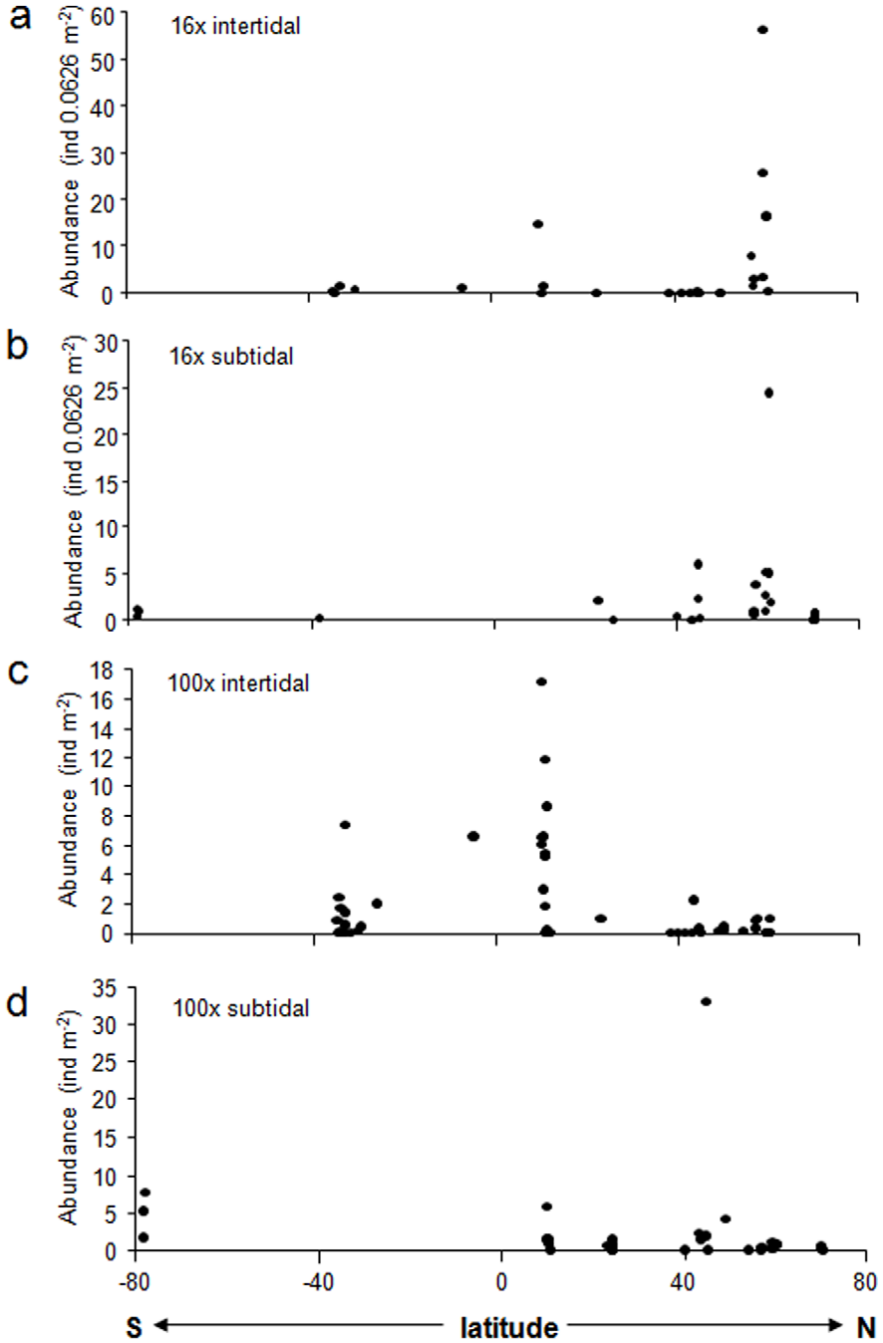
Spearman rank correlations between echinoderm abundance and latitude. Echinoderm abundances are from **a.** 16x intertidal collections, **b.** 16x subtidal collections, **c.** 100x intertidal collections, and **d.** 100x subtidal collections. See Tables 1 and 2 for correlation coefficients and significance levels.

**Figure 6:**
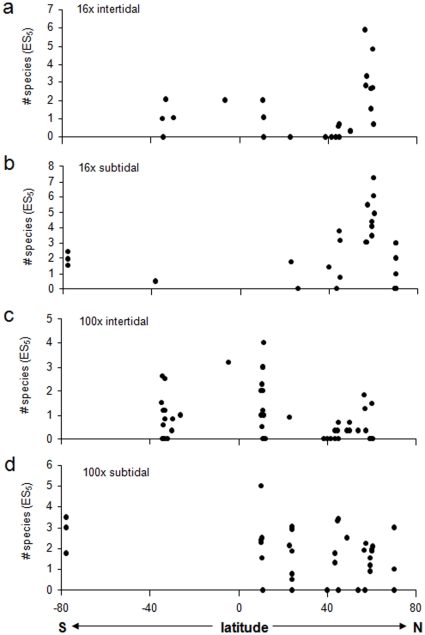
Spearman rank correlations between echinoderm species richness (based on estimated number of species ES_5_) and latitude. Echinoderm abundances are from **a.** 16x intertidal collections, **b.** 16x subtidal collections, **c.** 100x intertidal collections, and **d.** 100x subtidal collections. See Tables 1 and 2 for correlation coefficients and significance levels.

**Figure 7:**
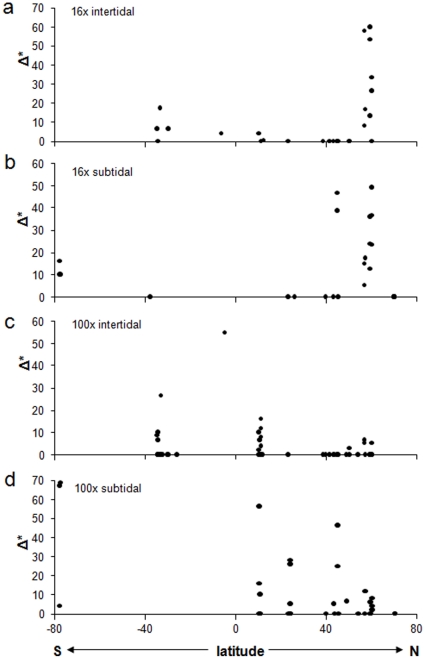
Spearman rank correlations between echinoderm taxonomic distinctness and latitude. Echinoderm abundances are from **a.** 16x intertidal collections, **b.** 16x subtidal collections, **c.** 100x intertidal collections, and **d.** 100x subtidal collections. See Tables 1 and 2 for correlation coefficients and significance levels.

Latitudinal trends were significant for correlations with abundance and ES_5_ in 16x intertidal assemblages (Table 2, Figs. 5a and 6a). In both cases correlations were positive, indicating higher abundance and species richness at higher northern latitudes with decreases towards lower latitudes and the southern hemisphere. The positive correlation of abundance with latitude was slightly reduced in correlation strength and was marginally not significant when abundance residuals were used (i.e., corrected for sampling effort) (Table 2). These latitudinal trends were likely driven mostly by the high values around 60° N, i.e. the Northeast Pacific region (also see Figs. 2a and 4a). None of the other correlations with latitude were significant (Table 2, Figs. 5,6,7), although the correlation between taxonomic distinctness and latitude for 16x intertidal assemblages was only marginally non-significant (Table 2, Fig. 7a). Also the correlations for both abundance and ES_5_ for 100x intertidal assemblages were marginally non-significant (Table 3). Visual inspection of these latter two correlation plots (Figs. 5c and 6c, respectively) indicated that instead of a continuous gradient across both hemispheres, there might be a peak at low latitudes (∼10–11°N) with declines towards higher latitudes at either side. Separate Spearman rank correlations for abundances from 10–60°N and for 11°N–34°S were both significant (rho = −0.550, p = 0.001 for 10–60°N; rho = 0.484, p = 0.007 for 11°N–34°S), confirming highest abundances at lower latitudes, i.e. in the Caribbean. Similarly, separate Spearman rank correlations of ES_5_ with latitude for the same latitudinal groups confirmed a significant decline in species richness from low to high northern latitudes (10–60°N: rho = −0.458, p = 0.005) while no significant decline into the southern hemisphere was observed (11°N–34°S), but data coverage in the southern hemisphere was low.

**Table 2:** Intertidal and subtidal echinoderm assemblages from 16x quadrat collections: Spearman Rank Correlation (rho) of abundance (N), residuals of abundance regressed against number of sampling quadrats (N_resid_), expected number of taxa ES_5_, and Taxonomic Distinctness index (Δ*) versus latitude (Lat).

	*intertidal (n = 26)*		*subtidal (n = 31)*	
	rho	p-value	rho	p-value
N vs Lat	0.463	0.017	−0.236	0.166
N_resid_ vs Lat	0.322	0.051	−0.317	0.059
ES_5_ vs Lat	0.422	0.032	−0.059	0.734
Δ* vs Lat	0.377	0.063	−0.306	0.094

Bold print indicates significant correlations at α≤0.05.

**Table 3:** Intertidal and subtidal echinoderm assemblages from 100x quadrat collections: Spearman Rank Correlation (rho) of abundance (N), residuals of abundance regressed against number of sampling quadrats (N_resid_), expected number of taxa ES_5_, and Taxonomic Distinctness index (Δ*) versus latitude (Lat).

	*intertidal (n = 52)*		*subtidal (n = 36)*	
	rho	p-value	rho	p-value
N vs Lat	−0.253	0.070	−0.215	0.208
N_resid_ vs Lat	−0.205	0.145	−0.280	0.115
ES_5_ vs Lat	−0.271	0.052	−0.284	0.093
Δ* vs Lat	−0.185	0.190	−0.290	0.086

### Correlation of echinoderm assemblages with environmental drivers

The highest correlation between the set of 14 environmental variables and 16x assemblage data for the four ecoregions Northeast Pacific, Northwest Atlantic, Caribbean and Alaskan Arctic occurred with a combination of three variables (rho = 0. 948): sea-surface temperature, inorganic pollution and nutrient contamination. The single variables sea-surface temperature and inorganic pollution each by itself yielded a correlation of rho = 0.894 (Table 4a). The combination of salinity, sea-surface temperature, chlorophyll *a* and primary production correlated strongly (rho = 0.900) with 100x assemblages for the six ecoregions Northeast Pacific, Northwest Atlantic, Caribbean, Indian Ocean African coast, Atlantic Ocean African coast and Alaskan Arctic. Among these variables, salinity alone yielded the highest coefficient with 100x ecoregional data (rho = 0.761, Table 4b). When chlorophyll *a* and primary production as variables were excluded from the 100x analysis because data derived from spectral ocean properties may be associated with large errors, especially in the nearshore system [55], overall correlation strength was reduced (rho = 0.771) driven by salinity and sea-surface temperature, with salinity remaining the most important variable (rho = 0.761).

**Table 4:** Spearman Rank Correlation (BEST Bio-Env analysis) between echinoderm community structure and 14 main environmental variables (see Table 1 and text for details and abbreviations).

a. 16x collectionsecoregions included	rho	best variable combination	best single variable
NEP, NWA, CAR, ARC	0.948	SST, INP, NUTC	SST (rho = 0.0.894)

b. 100x collectionsecoregions included	rho	best variable combination	best single variable
NEP, NWA, CAR, ARC, AAF, IAF	0.900	SAL, SST, CHA, PP	SAL (rho = 0.761)
Variables CHA and PP excluded	0.771	SAL, SST	SAL (rho = 0.761)

Analyses were based on Theta dissimilarity of taxa on the ecoregion scale correlated with environmental variables on the ecoregion scale for **A.** echinoderm assemblages from 16x quadrat collections; and **B.** echinoderm assemblages from 100x quadrat collections. Best variable combinations yielding the highest correlation coefficient are listed. In addition, correlation coefficients of the single best variable are listed.

## Discussion

Echinoderm diversity is typically higher in coastal regions than deeper waters [34], [59]; however, while echinoderms typically are a conspicuous and abundant component within intertidal and shallow subtidal habitats, they often are not overly diverse compared to other phyla [37], [60]–[62]. Similarly, species richness in our collections from nearshore rocky macroalgal habitats was low with typically only 1–5 species at those sites where echinoderms were present. Several of our sites did not contain any echinoderms (18 of 76), irrespective of site location or ecoregion. Despite low local diversity and abundance, echinoderms often are ecologically important in intertidal and shallow water systems, particularly as predators and grazers [63]–[65], emphasizing the need to consider their large-scale distribution and diversity patterns, such as latitudinal trends, and to identify environmental drivers that may influence these assemblages.

Even though the low overall echinoderm species richness found here is consistent with findings of other studies for intertidal and shallow subtidal habitats [60]–[62], we emphasize that overall species richness at any given site was certainly underestimated due to the sampling scheme used. The use of the standardized NaGISA sampling protocol [49] is useful for comparison of sample-based diversity among sites [51], also referred to as point diversity [66]–[67] or species density [51], but is not a reliable tool to comprehensively assess local (alpha) diversity within a community [68]–[69]. While it is not always obvious how a community would be defined for the assessment of alpha diversity [68], the 5–15 replicate quadrats per tidal range as collected here are not likely to inventory the entire community. Hence, data collected with the NaGISA protocol or similar standardized sampling designs will only represent a subset of the species occurring in a certain community, i.e., a subset of alpha diversity. The benefit of using this species density as a measure in large-scale comparisons is that there is no ambiguity of defining the scale of community as it exists for alpha diversity. Other benefits lie in the comparability of a standardized sampling effort and the practicality of how many replicate samples can feasibly be processed if large-scale coverage is the goal. The efficiency of quadrat sampling is dependent on the size and distribution patterns of the target organisms and may not be as effective in assessing patterns of highly patchily distributed taxa such as echinoderms [70]–[71]. The 100x quadrats (only organisms >2 cm collected) targeted larger-sized echinoderms, mostly adult asteroids and echinoids (see Fig. 3c and d) that typically are patchily distributed. In comparison, many small-sized ophiuroids and holothurians, and juvenile asteroids and echinoids were included in the collections from the 16x quadrats, which often occur in large densities. Therefore, the observed differences between assemblages with respect to different quadrat sizes are not surprising as the spatial patterns of diversity can be influenced by the scale at which those observations are made [9]–[10], [72]–[73]. We propose that visual assessments of echinoderm richness should rather be done with belt transects than 1×1 m quadrats because of the typically large size and patchy distribution of most echinoderm species.

### Large-scale trends in echinoderm assemblages

Abundance and species richness (ES_5_) in assemblages from the 100x intertidal collections followed the suggested generalized latitudinal gradient pattern [11], with highest values at low latitudes (Caribbean) and clines in both hemispheres towards higher latitudes. In the Caribbean, these assemblages consisted mainly of echinoids (see Fig. 3c). Similar peaks in sea urchin abundance and diversity at low latitudes have been found previously in regional-scale investigations in the Mediterranean [74]. The causes for this pattern are uncertain but may be related to a prevalence of thermophilic species in sea urchins [75], latitudinal differences in recruitment success [76]–[77] and salinity tolerance [78], competitive and predatory interactions [79]–[80], relief from predation due to overfishing [27], as well as high adaptability of sea urchins to environmental stress [81]–[82]. The latter is likely relaxed for subtidal communities because of generally more buffered physical environmental conditions in the subtidal [83], and may contribute to the non-significant abundance and diversity patterns in the 100x subtidal assemblages.

In contrast to the 100x intertidal assemblages, the 16x intertidal assemblages showed a gradient of highest abundance and richness at high northern latitudes with declines towards lower latitudes. These declines continued into the southern hemisphere, although sample coverage there was low and our patterns in the southern hemisphere have to be considered with care within a global context. Latitudinal trends observed here therefore are mainly driven by, and are most relevant for, the more intensively sampled northern hemisphere. This latitudinal decline in 16x intertidal echinoderm assemblage abundance and richness was mostly driven by high values in the Northeast Pacific, a highly productive and diverse ecoregion [62], [84]. Correlation strength of this latitudinal cline in 16x intertidal assemblages was moderate (0.32–0.46 for abundance and 0.42 for ES_5_, Table 2) but was comparable or even stronger than correlation strengths found in other studies observing marine latitudinal gradients (e.g., 0.13–0.39) [9]. Most notably, the latitudinal pattern of highest diversity in high northern latitudes (as observed for 16x intertidal assemblages) is not consistent with the postulated pattern of low latitudinal diversity peaks [11].

The lack of other global or truly large-scale diversity studies for echinoderms limits our comparisons to regional studies. For example, echinoderm diversity along the eastern Australian coast down to Tasmania decreased with increasing latitude [85], similar to what we observed for 100x intertidal echinoderms. In Australia this pattern was argued to be related mostly to the geological history of the continent, which favored continued immigration of new species from the tropics in Australia's north but led to progressive exclusion of cold-water species and survival of only few adaptive genera in the high-latitude south [85], [86]. Immigration in addition to vicariance has often been presented to explain higher diversity in tropical compared to temperate regions [87]–[89]. One would expect that as a result of such evolutionary patterns, tropical or low latitude regions should not only be more diverse on the species level but also on higher taxonomic levels, which effectively is a measure of taxonomic distinctness [90]. While we have no information on taxonomic distinctness for the echinoderm assemblages along the Australian coast [85], taxonomic distinctness in our study did not show significant trends with latitude for any of the assemblage types (16x and 100x, intertidal and subtidal) analyzed. However, while marginally not significant, taxonomic distinctness was higher in high northern latitudes (Northeast Pacific) in 16x intertidal assemblages (Fig. 7a, Table 2). Similarly, although for subtidal soft-bottom assemblages, taxonomic distinctness of asteroid assemblages across the Atlantic Ocean was highest at higher latitudes [59]. This may be an indication that at least some components of the cold-water adapted echinoderm fauna may derive from a larger number of higher taxonomic levels than at lower latitudes.

Even though we observed several strong latitudinal trends in the 16x intertidal and 100x intertidal echinoderm assemblages (Tables 2 and 3), some of these trends were strongly driven by particularly high abundances and/or species numbers in certain regions. In some cases these high values may be influenced by regional sampling effort. Although we corrected for sampling effort at each site, it remains that some regions were more intensively sampled than others. Higher regional sampling effort may contribute to the observation of higher abundance and diversity measures. For example, high sampling effort in the Northeast Pacific 16x intertidal coincided with high abundances and species richness. This is not a general pattern, however, as, for example, the large number of sites sampled for the 100x intertidal in the Northeast Pacific resulted in low abundance and species richness for that assemblage type (Figs. 2 and 4, respectively). Similarly, even though for the 16x subtidal assemblages the highest number of sites (11 sites) was sampled in the Arctic, abundances and species richness remained very low. Still, differences in regional sampling effort are likely to influence any latitudinal or other large-scale comparisons. Sufficient replication per latitude or latitudinal ranges and increased coverage of especially southern hemisphere coastlines will be needed for the evaluation of reliable global trends. Even though our data coverage is the most comprehensive currently available for echinoderms from nearshore rocky macroalgal habitats, we still lack sufficient coverage along many coastlines and latitudinal ranges.

It also is likely that overall latitudinal trends are interspersed with regional hotspots that disrupt, or go against, the main trend. Several ecoregions may be identified as hotspots for nearshore echinoderms in rocky habitats based on their overall high echinoderm abundance and species richness, e.g., in the Caribbean, the Northeast Pacific, the Northwest Atlantic, the Atlantic and Indian African coasts, and the Antarctic McMurdo Sound. It is possible that regional and local conditions act on top of latitudinal location and influence patterns in echinoderm assemblages. For example, Price et al. [59] found that shallow-water asteroid assemblages across the Atlantic Ocean were more distinct by geographic region and the level of isolation among regions than by latitude. In the Antarctic, echinoderm assemblages were more influenced by a combination of local and regional processes such as oceanographic conditions and iceberg scour intensity than by latitude [91]. We also observed relatively high diversity in subtidal echinoderm assemblages in the Antarctic, a region that is known to have high diversity for many taxa based on the long evolutionary isolation of the Southern Ocean, and high nutrient levels leading to high primary productivity [92]–[95]. In contrast, the low abundance and species richness we found in the Arctic region is consistent with other observations of a sharp decline in diversity in the Arctic compared to northern high latitudes, due to the severe ice impact and low food abundance in the Arctic [61], [96]–[100]. Among the ecoregions that may be hotspots, the Northeast Pacific, Northwest Atlantic, African Atlantic and Antarctic all are highly productive regions [40], [84], [101] providing rich food sources for nearshore echinoderms in rocky habitats [102]. Typically, this should also be reflected in high abundances, which we found in some echinoderm assemblages (esp. 16x intertidal and subtidal, Fig. 2) in the Northeast Pacific, and Northwest Atlantic, but not in the African Atlantic. In contrast, the Caribbean and African Indian Ocean are warm tropical/sub-tropical regimes where speciation rates and species radiation are high [88]. It seems likely that multiple, regionally different factors (e.g., productivity, evolutionary history) contribute to the high echinoderm diversity of these hotspots. It will be interesting in continued studies to add regions that are similar in general ecosystem characteristics to those we identified as hotspots here. For example, the southern Chilean fjords and the northern Norwegian fjords share many characteristics with regions in the Northwest Pacific examined here and would lend themselves for comparative studies. Overall, we see our data and latitudinal analyses as a first attempt of a global assessment of echinoderm distribution patterns that provides a baseline that can and should be tested in the future as more data become available.

### Environmental drivers of echinoderm assemblages

The identity of drivers of large-scale patterns in marine diversity patterns is often unexplored. While latitude can be an overall strong correlate, albeit, as we have shown here is not always consistent and predictable, latitude itself is likely a surrogate for some other underlying mechanisms. These mechanisms are typically not well understood. We used a correlative approach (BEST Bio-Env procedure) to analyze the importance of environmental drivers on large-scale echinoderm assemblage patterns, but it should be noted that this multivariate approach does not indicate directionality of a particular driver. Except for macroalgal biomass and substrate, all variables were extracted from large-scale databases and resolution did not match our site-specific sampling protocol in scale and may not reflect local, site-specific conditions. It has been shown that the scale of drivers, e.g. local versus regional versus large-scale effects, as well as their interactions can affect marine assemblage structure [103]. We have concentrated here on large-scale comparisons as variability among ecoregions is much larger compared to local scales [57]. Hence, despite the uncertainty of accuracy of environmental variables on the local scale, differences on large scales among ecosystems are more reliably represented [56]. It should be noted that other variables not included in our list are known to influence echinoderm assemblages and nearshore communities in general, e.g., sedimentation [104], exposure [72], [105], resource availability [106], and fisheries effects, potentially releasing echinoderms from predation pressure [27], [107].

The natural variables salinity, sea-surface temperature, chlorophyll *a* and primary productivity in addition to the anthropogenic drivers of inorganic pollution and nutrient contamination emerged as strongest correlates in our analyses with echinoderm assemblages on the ecoregional scale. Salinity could have physiological effects on echinoderm assemblages. Echinoderms are largely stenohaline and lack specific osmoregulatory organs and are sensitive to salinity fluctuations [108]. Salinity affects echinoderms both with hyposaline and hypersaline conditions, reducing larval dispersal and recruitment and leading to morphological deformations with reduced viability in adults [109]–[111]. Salinity was a particularly strong driver of 100x assemblages, possibly also because a larger number of ecoregions than for 16x assemblages was included with a large range of average salinities (31.4 in NEP–36.0 in CAR).

Sea-surface temperature, as a proxy for energy available to a system, has been suggested as an underpinning reason for observed diversity patterns [18], [112]. Temperature, however, affects organisms through metabolic and energetic processes, but does not directly influence diversity [113]. As such, taxa with different physiological adaptations and requirements may be differently influenced by temperature, possibly shaping the diversity of an assemblage in multiple ways. Intertidal assemblages collected with the NaGISA protocol also showed strong correlation with sea-surface temperature [57]. More detailed examinations of correlations between sea-surface temperature and intertidal and shallow-water overall community diversity found temporal variability in sea-surface temperature to affect diversity the most [101], [114]. Similarly, shallow-water asteroids in the Gulf of California also were driven mainly by variability in sea-surface temperature [115]. Generally, the lower seasonal variability in tropical regions allows more species to adapt than the large seasonal temperature extremes at higher latitudes, which could contribute to the high diversity we observed in the Caribbean and the Indian Ocean -African coast influenced by the warm Agulhas Current. This does not explain, however, the high diversity in the cold and highly seasonal regions of the Northeast Pacific and Northwest Atlantic and the constantly cold upwelling regions of the Atlantic Ocean -African coast (Benguela influence).

The productivity hypothesis states that species diversity should increase with increasing primary productivity [116]. Large-scale primary productivity and chlorophyll *a* patterns are particularly influenced by major current systems in the oceans, such as upwelling and cold-water currents [117], which could explain relatively high diversity and abundances in upwelling regions such as the Atlantic African coast. However, it is difficult to conclude latitudinal patterns from this, and the hypothesis has not been supported when tested for various upwelling systems [101]. Conversely, productivity and chlorophyll *a* concentration have been found to have negative relationships with asteroid diversity in the Gulf of California, possibly because of higher rates of larval mortality to predation in highly productive regions [115]. Different current and productivity systems may therefore influence different echinoderm taxa differently and not in a linear fashion with latitude.

Indices of inorganic pollution and nutrient contamination were the anthropogenic drivers most influencing echinoderm assemblages. Little is known on the effects of pollution on echinoderms, but it can lead to larval mortality and immunological deficiencies in echinoids [118]–[120] and pollution effects have been invoked to explain the deterioration of echinoderm diversity in the Gulf of Aqaba over the last decades [111]. It seems that although eutrophication (nutrient contamination) may first lead to an increase in species diversity, long-term effects on echinoderm assemblages are unknown in most cases [121].

In summary, a number of different variables appeared to structure echinoderm assemblages, painting a complex picture where no single environmental variable was the sole driver. It is likely that different echinoderm groups respond differently to environmental drivers, contributing to some of the disparate patterns we found for latitudinal gradients. This emphasizes that taxonomic relatedness of taxa (i.e., within the phylum Echinodermata) does not necessarily translate into ecological similarity, especially in a phylum exhibiting the large degree of adaptive radiation as in echinoderms. We suggest that information on life style, size, functional groups etc. of the investigated taxa as well as ecological drivers such as latitudinal differences in recruitment, predation, competition and interaction effects [122]–[124] should also be considered when analyzing and interpreting large-scale patterns in abundance and diversity.

## Supporting Information

Table S1 Echinoderm collection sites. Tidal height indicates the assemblage being collected (intertidal, subtidal) and quadrat sizes are 100x = 1 m2 and 16x = 0.0625 m2. Ecoregions are ARC  =  Alaska Arctic, NEP  =  north-east Pacific, CEP  =  central-east Pacific, WP  =  western Pacific, MED  =  Mediterranean, EUR  =  European Atlantic, NWA  =  north-west Atlantic, CAR  =  Caribbean, ASA  =  Atlantic South America, IAF  =  Indian Ocean Africa, AAF  =  Atlantic Ocean Africa, ANT  =  Antarctic McMurdo Sound. Environmental data were available for those sites marked with asterisks. Raw data for all sites can be obtained through the NaGISA website upon request: www.nagisa.coml.org.
(0.15 MB DOC)
