# Supplementary material for: Large-Scale Spatial Distribution Patterns of Echinoderms in Nearshore Rocky Habitats
Source: PLoS One. 2010 Nov 5;5(11):e13845. doi: 10.1371/journal.pone.0013845 (PMC2974624; doi:10.1371/journal.pone.0013845)
Supplement: Table S1 — Echinoderm collection sites. Tidal height indicates the assemblage being collected (intertidal, subtidal) and quadrat sizes are 100x = 1 m2 and 16x = 0.0625 m2. Ecoregions are ARC = Alaska Arctic, NEP = north-east Pacific, CEP = central-east Pacific, WP = western Pacific, MED = Mediterranean, EUR = European Atlantic, NWA = north-west Atlantic, CAR = Caribbean, ASA = Atlantic South America, IAF = Indian Ocean Africa, AAF = Atlantic Ocean Africa, ANT = Antarctic McMurdo Sound. Environmental data were available for those sites marked with asterisks. Raw data for all sites can be obtained through the NaGISA website upon request: www.nagisa.coml.org. (0.15 MB DOC) [file pone.0013845.s001.doc]

**Supplementary results**

**Large-scale Spatial Distribution Patterns in Echinoderms in Nearshore Rocky Habitats**

Katrin Iken^*^, Brenda Konar, Lisandro Benedetti-Cecchi, Juan José Cruz-Motta, Ann Knowlton, Gerhard Pohle, Angela Mead, Patricia Miloslavich, Melisa Wong, Thomas Trott, Nova Mieszkowska, Rafael Riosmena-Rodriguez, Laura Airoldi, Edward Kimani, Yoshihisa Shirayama, Simonetta Fraschetti, Manuel Ortiz-Touzet, Angelica Silva

* Corresponding author: iken@ims.uaf.edu

**Table of contents**

1. **Supplementary Table S1:** Echinoderm collection sites. Tidal height indicates the assemblage being collected (intertidal, subtidal) and quadrat sizes are 100x = 1 m^2^ and 16x = 0.0625 m^2^. Ecoregions are ARC = Alaska Arctic, NEP = north-east Pacific, CEP = central-east Pacific, WP = western Pacific, MED = Mediterranean, EUR = European Atlantic, NWA = north-west Atlantic, CAR = Caribbean, ASA = Atlantic South America, IAF = Indian Ocean Africa, AAF = Atlantic Ocean Africa, ANT = Antarctic McMurdo Sound. Environmental data were available for those sites marked with asterisks. Raw data for all sites can be obtained through the NaGISA website upon request: [www.nagisa.coml.org](http://www.nagisa.coml.org/).

**Supplementary Table S1**

| **Site** | **Year** | **Area** | **Country** | **Ocean** | **Tidal region** | **Quadrat size** | **Lat** | **Long** | **ecoregion** |
| --- | --- | --- | --- | --- | --- | --- | --- | --- | --- |
| * DS11 | 2006 | Alaska/Boulder Patch | USA | Arctic | subtidal | 100x, 16x | 70.3220 | -147.5790 | ARC |
| * E1 | 2006 | Alaska/Boulder Patch | USA | Arctic | subtidal | 16x | 70.3150 | -147.7320 | ARC |
| * E2 | 2006 | Alaska/Boulder Patch | USA | Arctic | subtidal | 16x | 70.3180 | -147.7150 | ARC |
| * E3 | 2006 | Alaska/Boulder Patch | USA | Arctic | subtidal | 100x, 16x | 70.3250 | -147.6490 | ARC |
| * W1 | 2006 | Alaska/Boulder Patch | USA | Arctic | subtidal | 16x | 70.3700 | -147.8730 | ARC |
| * W2 | 2006 | Alaska/Boulder Patch | USA | Arctic | subtidal | 16x | 70.3700 | -147.8600 | ARC |
| * W3 | 2006 | Alaska/Boulder Patch | USA | Arctic | subtidal | 100x, 16x | 70.3760 | -147.7940 | ARC |
| * DS4 | 2007 | Alaska/Camden Bay | USA | Arctic | subtidal | 16x | 70.0318 | -145.2688 | ARC |
| * DSC | 2005 | Alaska/Camden Bay | USA | Arctic | subtidal | 16x | 70.0247 | -145.2535 | ARC |
| * DSE | 2005 | Alaska/Camden Bay | USA | Arctic | subtidal | 16x | 70.0258 | -145.2588 | ARC |
| * DSW | 2007 | Alaska/Camden Bay | USA | Arctic | subtidal | 16x | 70.0245 | -145.2576 | ARC |
| * Ridley Island | 2007 | British Columbia/Prince Rupert | Canada | Pacific | intertidal, subtidal | 100x | 54.2207 | -130.3294 | NEP |
| * Bath Island | 2006 | British Columbia/Vancouver | Canada | Pacific | intertidal, subtidal | 100x | 49.1440 | -123.6720 | NEP |
| * Cohen Island | 2003 | Alaska/Kachemak Bay | USA | Pacific | Intertidal, subtidal | 100x, 16x | 59.5472 | -151.5472 | NEP |
| * Elephant Island | 2003 | Alaska/Kachemak Bay | USA | Pacific | intertidal, subtidal | 100x, 16x | 59.5472 | -151.5139 | NEP |
| * Outside Beach | 2003 | Alaska/Kachemak Bay | USA | Pacific | intertidal, subtidal | 100x, 16x | 59.4736 | -151.8942 | NEP |
| * Akhiok Bay | 2003 | Alaska/Kodiak Island | USA | Pacific | intertidal, subtidal | 100x, 16x | 56.9465 | -154.1293 | NEP |
| * Old Harbor | 2003 | Alaska/Kodiak Island | USA | Pacific | intertidal, subtidal | 100x, 16x | 57.1567 | -153.3887 | NEP |
| * Uyak Bay | 2003 | Alaska/Kodiak Island | USA | Pacific | intertidal, subtidal | 100x, 16x | 57.5736 | -154.1119 | NEP |
| * Green Island | 2003 | Alaska/Prince William Sound | USA | Pacific | intertidal, subtidal | 100x, 16x | 60.3000 | -147.4122 | NEP |
| * Knight Island | 2003 | Alaska/Prince William Sound | USA | Pacific | intertidal, subtidal | 100x, 16x | 60.4844 | -147.7356 | NEP |
| * Montague Island | 2003 | Alaska/Prince William Sound | USA | Pacific | intertidal, subtidal | 100x, 16x | 60.3908 | -147.1217 | NEP |
| El Sauzoso | 2009 | Bahia de La Paz | Mexico | Pacific | subtidal | 100x | 24.3117 | -110.6397 | CEP |
| Botafuego | 2009 | Bahia de La Paz | Mexico | Pacific | subtidal | 100x | 24.7566 | -110.6725 | CEP |
| El Coyote | 2009 | Bahia de La Paz | Mexico | Pacific | subtidal | 100x | 24.6994 | -110.6997 | CEP |
| El Saladito | 2009 | Bahia de La Paz | Mexico | Pacific | subtidal | 100x | 24.4398 | -110.6877 | CEP |
| El Quelele | 2009 | Bahia de La Paz | Mexico | Pacific | subtidal | 100x | 24.2044 | -110.5356 | CEP |
| Isla San Pedro | 2009 | Bahia de La Paz | Mexico | Pacific | subtidal | 100x | 28.3790 | -112.3192 | CEP |
| * Kesen-numa | 2006 | Tohoku | Japan | Pacific | intertidal | 16x | 38.8968 | 141.6250 | WP |
| * Sakamoto | 2008 | Miyagi | Japan | Pacific | intertidal | 100x | 38.6455 | 141.4774 | WP |
| **Site** | **Year** | **Area** | **Country** | **Ocean** | **Tidal region** | **Quadrat size** | **Lat** | **Long** | **ecoregion** |
| Maenohama | 2006 | Akajima | Japan | Pacific | subtidal | 16x | 26.1865 | 127.2806 | WP |
| * Hon Chong | 2009 | Nha Trang | Vietnam | Pacific | intertidal | 100x | 12.2714 | 109.2067 | WP |
| * Torre del Serpe | 2006 | Apulia/Otranto | Italy | Mediterranean | intertidal, subtidal | 100x inter  16x sub | 40.1447 | 18.5060 | MED |
| Conero Riviera | 2006 | Conero Riviera | Italy | Mediterranean | intertidal, subtidal | 100x | 43.5481 | 13.6281 | MED |
| Calafuria | 2006 | Tuscany/Livorno | Italy | Mediterranean | intertidal, subtidal | 100x, 16x | 43.4725 | 10.3329 | MED |
| Viana | 2006 | Viana do Castello | Portugal | Atlantic | intertidal | 100x, 16x | 41.6967 | -8.8531 | EUR |
| * Looe | 2007 | Cornwall | UK | Atlantic | intertidal | 100x, 16x | 50.3408 | -4.4595 | EUR |
| * Batten Bay | 2007 | Devon | UK | Atlantic | intertidal | 100x, 16x | 50.3566 | -4.1269 | EUR |
| * Simpsons Island | 2008 | Passamaquoddy Bay | Canada | Atlantic | intertidal, subtidal | 100x, 16x | 45.0038 | -66.9136 | NWA |
| * Canso | 2008 | Northeastern Shore | Canada | Atlantic | intertidal, subtidal | 100x, 16x | 45.3267 | -60.9633 | NWA |
| * Birch Island | 2007 | Maine/Cobscook Bay | USA | Atlantic | intertidal, subtidal | 100x, 16x | 44.8711 | -67.1493 | NWA |
| * Playa de 16 | 2006 | Miramar | Cuba | Atlantic | intertidal, subtidal | 100x, 16x | 23.1283 | -82.4222 | CAR |
| * Fort Granby | 2007 | Caribbean | Trinidad | Atlantic | intertidal | 100x, 16x | 11.1862 | -60.6606 | CAR |
| Punta Yapascua | 2007 | Carabobo/Patanemo | Venezuela | Atlantic | intertidal | 16x | 10.4750 | -67.9015 | CAR |
| * Piedra Ahogada | 2008 | Mochima | Venezuela | Atlantic | intertidal, subtidal | 100x | 10.3891 | -64.3478 | CAR |
| * Punta Cruz | 2008 | Mochima | Venezuela | Atlantic | intertidal, subtidal | 100x | 10.3957 | -54.3675 | CAR |
| * Punta Tigrillo | 2008 | Mochima | Venezuela | Atlantic | intertidal, subtidal | 100x | 10.3804 | -64.3951 | CAR |
| * Boca Seca | 2008 | Morrocoy | Venezuela | Atlantic | intertidal, subtidal | 100x | 10.8319 | -68.2408 | CAR |
| * Cayo Mero | 2008 | Morrocoy | Venezuela | Atlantic | intertidal, subtidal | 100x, 16x | 10.8198 | -68.2477 | CAR |
| * Playa Caimán | 2008 | Morrocoy | Venezuela | Atlantic | intertidal, subtidal | 100x | 10.8517 | -68.2363 | CAR |
| Centro San José | 2008 | San José de la Costa | Venezuela | Atlantic | intertidal | 100x | 11.4385 | -68.8377 | CAR |
| * Oeste San José | 2008 | San José de la Costa | Venezuela | Atlantic | intertidal | 100x | 11.4465 | -68.8851 | CAR |
| Sur San José | 2008 | San José de la Costa | Venezuela | Atlantic | intertidal | 100x | 11.4411 | -68.8292 | CAR |
| Centro San Juan | 2008 | San Juan de los Cayos | Venezuela | Atlantic | intertidal | 100x | 11.1842 | -68.4027 | CAR |
| Norte San Juan | 2008 | San Juan de los Cayos | Venezuela | Atlantic | intertidal | 100x | 11.1896 | -68.4094 | CAR |
| Sur San Juan | 2008 | San Juan de los Cayos | Venezuela | Atlantic | intertidal | 100x | 11.1802 | -68.3974 | CAR |
| Playa Chica | 2008 | Mar del Plata | Argentina | Atlantic | subtidal | 16x | -38.0200 | -57.5200 | n/d |
| * Pearly Beach | 2007 | Bantams Klip | South Africa | Atlantic | intertidal | 100x, 16x | -34.5848 | 19.4572 | AAF |
| * Cape Point | 2009 | False Bay | South Africa | Atlantic | intertidal | 100x | -34.3202 | 18.4611 | AAF |
| * Oudekraal | 2007 | False Bay | South Africa | Atlantic | intertidal | 100x | -33.9822 | 18.3550 | AAF |
| * Kreef Bay | 2008 | Langebaan | South Africa | Atlantic | intertidal | 100x, 16x | -33.1429 | 17.9821 | AAF |
| * Brasil | 2007 | Port Nollath | South Africa | Atlantic | intertidal | 100x, 16x | -29.7177 | 17.0582 | AAF |
| * Lamberts Bay | 2007 | Port Nollath | South Africa | Atlantic | intertidal | 100x | -32.0964 | 18.3016 | AAF |
| * Skulpfontein | 2007 | Port Nollath | South Africa | Atlantic | intertidal | 100x | -30.1890 | 17.2242 | AAF |
| **Site** | **Year** | **Area** | **Country** | **Ocean** | **Tidal region** | **Quadrat size** | **Lat** | **Long** | **ecoregion** |
| * Oysterbay | 2007 | Thyspunt | South Africa | Indian | intertidal | 100x, 16x | -34.1988 | 24.7979 | IAF |
| * Bat Cave Rocks | 2007 | East London | South Africa | Indian | intertidal | 100x | -33.0028 | 27.9414 | IAF |
| * Nahoon Reef | 2008 | East London | South Africa | Indian | intertidal | 100x | -32.9833 | 27.9494 | IAF |
| * Bronza Bay | 2007 | East London | South Africa | Indian | intertidal | 100x | -32.9946 | 27.9494 | IAF |
| * Goukamma | 2008 | Port Elizabeth | South Africa | Indian | intertidal | 100x | -34.0786 | 22.9513 | IAF |
| * Humewood | 2007 | Port Elizabeth | South Africa | Indian | intertidal | 100x | -33.9812 | 25.6551 | IAF |
| * Reef Bay | 2007 | Port Elizabeth | South Africa | Indian | intertidal | 100x | -34.0293 | 25.6846 | IAF |
| Wasini Island | 2008 | Shimoni | Kenya | Indian | intertidal | 100x | -4.6364 | 39.3579 | IAF |
| Barrara Vermelha | 2008 | Inhaca | Mozambique | Indian | intertidal | 100x | -26.0194 | 32.9017 | IAF |
| Dayton's Wall | 2008 | Ross Sea/McMurdo Sound | Antarctica | Southern | subtidal | 100x, 16x | -77.8532 | 166.6615 | ANT |
| Evan's Wall | 2008 | Ross Sea/McMurdo Sound | Antarctica | Southern | subtidal | 100x, 16x | -77.6569 | 166.5183 | ANT |
| Jetty | 2008 | Ross Sea/McMurdo Sound | Antarctica | Southern | subtidal | 100x, 16x | -77.8513 | 166.6645 | ANT |
